# Diaqua­bis{2-[5-(2-pyrid­yl)-2*H*-tetra­zol-2-yl]acetato-κ^2^ *N* ^4^,*N* ^5^}zinc(II)

**DOI:** 10.1107/S1600536809023940

**Published:** 2009-06-27

**Authors:** Bo Wang

**Affiliations:** a Ordered Matter Science Research Center, College of Chemistry and Chemical Engineering, Southeast University, Nanjing 210096, People’s Republic of China

## Abstract

The title compound, [Zn(C_8_H_6_N_5_O_2_)_2_(H_2_O)_2_], was synthesized by hydro­thermal reaction of ZnBr_2_ with 2-[5-(2-pyrid­yl)-2*H*-tetra­zol-2-yl]acetic acid. The Zn^II^ atom lies on an inversion center in a distorted octa­hedral environment with two planar *trans*-related *N*,*N*′-chelating 2-[5-(2-pyrid­yl)-2*H*-tetra­zol-2-yl]acetic acid ligands in the equatorial plane and two water mol­ecules in the axial positions. In the crystal, O—H⋯O hydrogen bonds generate an infinite three-dimensional network.

## Related literature

For the chemisty of tetra­zoles, see: Fu *et al.* (2008 ▶); Dai & Fu (2008 ▶); Wang *et al.* (2005 ▶); Wen (2008 ▶); Wittenberger & Donner (1993 ▶).
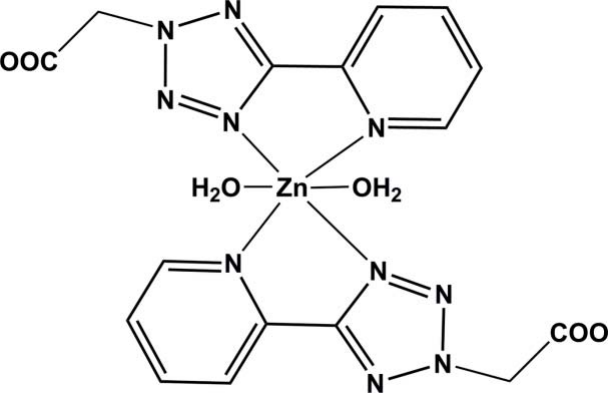


## Experimental

### 

#### Crystal data

[Zn(C_8_H_6_N_5_O_2_)_2_(H_2_O)_2_]
*M* *_r_* = 509.76
Monoclinic, 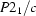

*a* = 7.6407 (15) Å
*b* = 8.2583 (17) Å
*c* = 15.155 (3) Å
β = 97.17 (3)°
*V* = 948.8 (3) Å^3^
*Z* = 2
Mo *K*α radiation
μ = 1.36 mm^−1^
*T* = 298 K
0.35 × 0.25 × 0.20 mm

#### Data collection

Rigaku Mercury2 diffractometer
Absorption correction: multi-scan (*CrystalClear*; Rigaku, 2005 ▶) *T*_min_ = 0.762, *T*_max_ = 0.841 (expected range = 0.690–0.762)
9600 measured reflections
2177 independent reflections
1984 reflections with *I* > 2σ(*I*)
*R*_int_ = 0.031

#### Refinement

*R*[*F* ^2^ > 2σ(*F* ^2^)] = 0.027
*wR*(*F* ^2^) = 0.075
*S* = 1.11
2177 reflections
151 parameters
H-atom parameters constrained
Δρ_max_ = 0.25 e Å^−3^
Δρ_min_ = −0.41 e Å^−3^

### 

Data collection: *CrystalClear* (Rigaku, 2005 ▶); cell refinement: *CrystalClear*; data reduction: *CrystalClear*; program(s) used to solve structure: *SHELXS97* (Sheldrick, 2008 ▶); program(s) used to refine structure: *SHELXL97* (Sheldrick, 2008 ▶); molecular graphics: *ORTEPIII* (Burnett & Johnson, 1996 ▶), *ORTEP-3 for Windows* (Farrugia, 1997 ▶) and *XP* in *SHELXTL* (Sheldrick, 2008 ▶); software used to prepare material for publication: *SHELXTL*.

## Supplementary Material

Crystal structure: contains datablocks I, global. DOI: 10.1107/S1600536809023940/dn2467sup1.cif

Structure factors: contains datablocks I. DOI: 10.1107/S1600536809023940/dn2467Isup2.hkl

Additional supplementary materials:  crystallographic information; 3D view; checkCIF report

## Figures and Tables

**Table 1:** Hydrogen-bond geometry (Å, °)

*D*—H⋯*A*	*D*—H	H⋯*A*	*D*⋯*A*	*D*—H⋯*A*
O1*W*—H1*WB*⋯O1^i^	0.85	1.85	2.6891 (19)	172
O1*W*—H1*WA*⋯O2^ii^	0.85	1.80	2.6365 (17)	169

Symmetry codes: (i) 
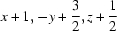
; (ii) 
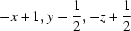
.

**Fig. 1.**
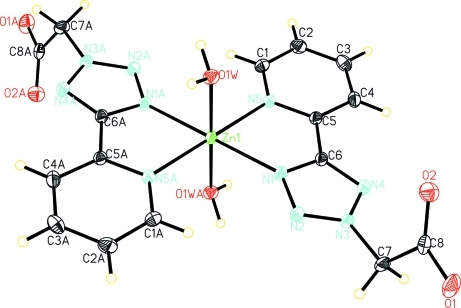
Molecular view of the title compound with the atomic numbering scheme. Displacement ellipsoids are drawn at the 30% probability level.[Symmetry codes: (i) -x+1, -y+1, -z+1]

**Fig. 2.**
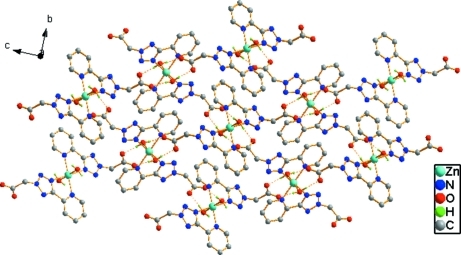
The crystal packing of the title compound viewed along the a axis showing the three dimensionnal hydrogen bondings network (dashed line). Hydrogen atoms not involved in hydrogen bonding have been omitted for clarity.

## supplementary crystallographic information

### Comment

The tetrazole functional group has found a wide range of applications in coordination chemistry as ligands, in medicinal chemistry as a metabolically stable surrogate for a carboxylic acid group, and in materials science as high density energy materials(Wang *et al.*, 2005; Fu *et al.*, 2008; Wittenberger *et al.*,1993). We report here the crystal structure of the title compound, Bis[2-(5-(pyridin-2-yl)-2*H*-tetrazol-2-yl)acetic-*K*^2^N^1^,N^2^]Zinc(II).

In the title compound, the Zn^II^ atom lies on an inversion center. The distorted octahedral Zn^II^ environment contains two planar trans-related N,N-chelating 2-(5-(pyridin-2-yl)-2*H*-tetrazol-2-yl)acetic acid ligands in the equatorial plane and two water ligands in the axial positions. The pyridine and tetrazole rings are nearly coplanar and only twisted from each other by a dihedral angle of 7.06 ( 1 )°. The geometric parameters of the tetrazole rings are comparable to those in related molecules (Wittenberger *et al.*, 1993; Dai & Fu 2008; Wen 2008).

The O atoms from water molecules are involved in intermolecular O—H···O hydrogen bonds building up an infinite three-dimensional network (Table 1 and Fig.2).

### Experimental

A mixture of 2-(5-(pyridin-2-yl)-2*H*-tetrazol-2-yl)acetic acid (0.2 mmol), ZnBr_2_ (0.4 mmol), distilled water (1 ml) and a few drops of ethanol sealed in a glass tube was maintained at 110 °C. Colorless block crystals suitable for X-ray analysis were obtained after 3 days.

### Refinement

All H atoms attached to C atoms were fixed geometrically and treated as riding with C-H = 0.93 Å (aromatic) and 0.97 Å (methylene) with *U*_iso_(H) = 1.2*U*_eq_(C). H atoms of water molecule located in difference Fourier maps and freely refined using restraints (O-H= 0.85Å and H···H= 1.39Å with *U*ĩso~(H) = 1.5*U*~eq~(O). In the last stage of refinement they were treated as riding on the O atom.

### Crystal data

**Table:**

[Zn(C_8_H_6_N_5_O_2_)_2_(H_2_O)_2_]	*F*(000) = 520
*M**_r_* = 509.76	*D*_x_ = 1.784 Mg m^−^^3^
Monoclinic, *P*2_1_/*c*	Mo *K*α radiation, λ = 0.71073 Å
Hall symbol: -P 2ybc	Cell parameters from 1984 reflections
*a* = 7.6407 (15) Å	θ = 3.6–27.5°
*b* = 8.2583 (17) Å	µ = 1.36 mm^−^^1^
*c* = 15.155 (3) Å	*T* = 298 K
β = 97.17 (3)°	Block, colorless
*V* = 948.8 (3) Å^3^	0.35 × 0.25 × 0.20 mm
*Z* = 2	

### Data collection

**Table:**

Rigaku Mercury2 diffractometer	2177 independent reflections
Radiation source: fine-focus sealed tube	1984 reflections with *I* > 2σ(*I*)
graphite	*R*_int_ = 0.031
Detector resolution: 13.6612 pixels mm^-1^	θ_max_ = 27.5°, θ_min_ = 3.6°
CCD profile fitting scans	*h* = −9→9
Absorption correction: multi-scan (*CrystalClear*; Rigaku, 2005)	*k* = −10→10
*T*_min_ = 0.762, *T*_max_ = 0.841	*l* = −19→19
9600 measured reflections	

### Refinement

**Table:**

Refinement on *F*^2^	Primary atom site location: structure-invariant direct methods
Least-squares matrix: full	Secondary atom site location: difference Fourier map
*R*[*F*^2^ > 2σ(*F*^2^)] = 0.027	Hydrogen site location: inferred from neighbouring sites
*wR*(*F*^2^) = 0.075	H-atom parameters constrained
*S* = 1.11	*w* = 1/[σ^2^(*F*_o_^2^) + (0.0393*P*)^2^ + 0.3221*P*] where *P* = (*F*_o_^2^ + 2*F*_c_^2^)/3
2177 reflections	(Δ/σ)_max_ < 0.001
151 parameters	Δρ_max_ = 0.25 e Å^−^^3^
0 restraints	Δρ_min_ = −0.41 e Å^−^^3^

### Special details

**Table:**

Geometry. All esds (except the esd in the dihedral angle between two l.s. planes) are estimated using the full covariance matrix. The cell esds are taken into account individually in the estimation of esds in distances, angles and torsion angles; correlations between esds in cell parameters are only used when they are defined by crystal symmetry. An approximate (isotropic) treatment of cell esds is used for estimating esds involving l.s. planes.
Refinement. Refinement of F^2^ against ALL reflections. The weighted R-factor wR and goodness of fit S are based on F^2^, conventional R-factors R are based on F, with F set to zero for negative F^2^. The threshold expression of F^2^ > 2sigma(F^2^) is used only for calculating R-factors(gt) etc. and is not relevant to the choice of reflections for refinement. R-factors based on F^2^ are statistically about twice as large as those based on F, and R- factors based on ALL data will be even larger.

### Fractional atomic coordinates and isotropic or equivalent isotropic displacement parameters (Å^2^)

**Table:**

	*x*	*y*	*z*	*U*_iso_*/*U*_eq_	
Zn1	0.5000	0.5000	0.5000	0.02160 (10)	
N1	0.32018 (18)	0.61053 (17)	0.39557 (9)	0.0230 (3)	
O1W	0.72023 (15)	0.59206 (14)	0.44676 (8)	0.0267 (3)	
H1WA	0.7564	0.5199	0.4135	0.040*	
H1WB	0.8013	0.6233	0.4865	0.040*	
N5	0.45085 (17)	0.73160 (16)	0.55431 (9)	0.0212 (3)	
O1	−0.0465 (2)	0.79027 (18)	0.07872 (9)	0.0417 (3)	
N2	0.22867 (19)	0.57379 (18)	0.31894 (9)	0.0253 (3)	
O2	0.16960 (18)	0.89846 (16)	0.17318 (8)	0.0353 (3)	
N3	0.11283 (18)	0.68988 (17)	0.30393 (9)	0.0238 (3)	
C5	0.3275 (2)	0.82286 (19)	0.50739 (10)	0.0211 (3)	
C6	0.2532 (2)	0.74873 (19)	0.42345 (10)	0.0213 (3)	
N4	0.12076 (19)	0.80232 (17)	0.36653 (9)	0.0260 (3)	
C1	0.5300 (2)	0.7902 (2)	0.63096 (11)	0.0275 (4)	
H1	0.6149	0.7271	0.6643	0.033*	
C4	0.2795 (2)	0.9734 (2)	0.53507 (12)	0.0281 (4)	
H4	0.1925	1.0336	0.5013	0.034*	
C2	0.4912 (3)	0.9404 (2)	0.66288 (12)	0.0320 (4)	
H2	0.5501	0.9787	0.7162	0.038*	
C3	0.3640 (3)	1.0324 (2)	0.61438 (13)	0.0330 (4)	
H3	0.3349	1.1339	0.6348	0.040*	
C7	−0.0122 (2)	0.6946 (2)	0.22339 (11)	0.0303 (4)	
H7A	−0.1255	0.7307	0.2384	0.036*	
H7B	−0.0273	0.5858	0.1996	0.036*	
C8	0.0445 (2)	0.8059 (2)	0.15154 (11)	0.0258 (3)	

### Atomic displacement parameters (Å^2^)

**Table:**

	*U*^11^	*U*^22^	*U*^33^	*U*^12^	*U*^13^	*U*^23^
Zn1	0.02190 (16)	0.02041 (15)	0.02109 (15)	0.00495 (9)	−0.00280 (10)	−0.00076 (9)
N1	0.0233 (7)	0.0239 (7)	0.0206 (6)	0.0030 (5)	−0.0022 (5)	0.0012 (5)
O1W	0.0250 (6)	0.0283 (6)	0.0263 (6)	0.0004 (5)	0.0008 (5)	−0.0052 (5)
N5	0.0203 (6)	0.0205 (6)	0.0221 (7)	0.0006 (5)	−0.0002 (5)	0.0001 (5)
O1	0.0499 (8)	0.0481 (9)	0.0238 (6)	0.0109 (7)	−0.0081 (6)	0.0047 (6)
N2	0.0269 (7)	0.0258 (7)	0.0213 (7)	0.0009 (6)	−0.0041 (5)	0.0027 (5)
O2	0.0390 (7)	0.0371 (7)	0.0304 (7)	−0.0054 (6)	0.0071 (5)	0.0062 (6)
N3	0.0234 (7)	0.0250 (7)	0.0211 (7)	−0.0008 (5)	−0.0043 (5)	0.0048 (5)
C5	0.0198 (7)	0.0216 (7)	0.0217 (8)	−0.0003 (6)	0.0018 (6)	0.0029 (6)
C6	0.0211 (7)	0.0211 (7)	0.0214 (8)	0.0005 (6)	0.0012 (6)	0.0044 (6)
N4	0.0254 (7)	0.0257 (7)	0.0253 (7)	0.0031 (6)	−0.0031 (5)	0.0032 (6)
C1	0.0249 (8)	0.0301 (9)	0.0258 (8)	−0.0007 (7)	−0.0034 (7)	−0.0010 (7)
C4	0.0293 (9)	0.0231 (8)	0.0317 (9)	0.0049 (7)	0.0035 (7)	0.0030 (7)
C2	0.0362 (10)	0.0319 (9)	0.0270 (9)	−0.0047 (8)	0.0002 (7)	−0.0069 (7)
C3	0.0430 (11)	0.0229 (8)	0.0342 (10)	0.0004 (7)	0.0089 (8)	−0.0060 (7)
C7	0.0284 (9)	0.0334 (9)	0.0254 (8)	−0.0047 (7)	−0.0109 (7)	0.0067 (7)
C8	0.0292 (8)	0.0260 (8)	0.0217 (8)	0.0103 (7)	0.0012 (6)	0.0034 (6)

### Geometric parameters (Å, °)

**Table:**

Zn1—O1W	2.0974 (13)	N3—N4	1.324 (2)
Zn1—O1W^i^	2.0974 (13)	N3—C7	1.453 (2)
Zn1—N5	2.1340 (14)	C5—C4	1.377 (2)
Zn1—N5^i^	2.1340 (14)	C5—C6	1.461 (2)
Zn1—N1^i^	2.1640 (14)	C6—N4	1.321 (2)
Zn1—N1	2.1640 (14)	C1—C2	1.377 (3)
N1—N2	1.3142 (19)	C1—H1	0.9300
N1—C6	1.341 (2)	C4—C3	1.380 (3)
O1W—H1WA	0.8486	C4—H4	0.9300
O1W—H1WB	0.8480	C2—C3	1.373 (3)
N5—C1	1.332 (2)	C2—H2	0.9300
N5—C5	1.339 (2)	C3—H3	0.9300
O1—C8	1.235 (2)	C7—C8	1.529 (2)
N2—N3	1.305 (2)	C7—H7A	0.9700
O2—C8	1.236 (2)	C7—H7B	0.9700
			
O1W—Zn1—O1W^i^	180.0	N5—C5—C4	122.90 (15)
O1W—Zn1—N5	90.67 (5)	N5—C5—C6	113.44 (14)
O1W^i^—Zn1—N5	89.33 (5)	C4—C5—C6	123.65 (15)
O1W—Zn1—N5^i^	89.33 (5)	N4—C6—N1	111.77 (14)
O1W^i^—Zn1—N5^i^	90.67 (5)	N4—C6—C5	127.65 (15)
N5—Zn1—N5^i^	180.000 (1)	N1—C6—C5	120.58 (14)
O1W—Zn1—N1^i^	88.14 (5)	C6—N4—N3	101.29 (13)
O1W^i^—Zn1—N1^i^	91.86 (5)	N5—C1—C2	122.66 (16)
N5—Zn1—N1^i^	102.84 (5)	N5—C1—H1	118.7
N5^i^—Zn1—N1^i^	77.16 (5)	C2—C1—H1	118.7
O1W—Zn1—N1	91.86 (5)	C5—C4—C3	118.07 (17)
O1W^i^—Zn1—N1	88.14 (5)	C5—C4—H4	121.0
N5—Zn1—N1	77.16 (5)	C3—C4—H4	121.0
N5^i^—Zn1—N1	102.84 (5)	C3—C2—C1	118.66 (17)
N1^i^—Zn1—N1	180.0	C3—C2—H2	120.7
N2—N1—C6	107.04 (13)	C1—C2—H2	120.7
N2—N1—Zn1	140.18 (11)	C2—C3—C4	119.59 (17)
C6—N1—Zn1	111.18 (10)	C2—C3—H3	120.2
Zn1—O1W—H1WA	108.1	C4—C3—H3	120.2
Zn1—O1W—H1WB	112.8	N3—C7—C8	113.54 (14)
H1WA—O1W—H1WB	111.8	N3—C7—H7A	108.9
C1—N5—C5	118.12 (14)	C8—C7—H7A	108.9
C1—N5—Zn1	125.41 (11)	N3—C7—H7B	108.9
C5—N5—Zn1	116.46 (11)	C8—C7—H7B	108.9
N3—N2—N1	105.00 (13)	H7A—C7—H7B	107.7
N2—N3—N4	114.90 (13)	O1—C8—O2	129.21 (17)
N2—N3—C7	121.74 (15)	O1—C8—C7	113.30 (16)
N4—N3—C7	123.35 (14)	O2—C8—C7	117.48 (15)

Symmetry codes: (i) −*x*+1, −*y*+1, −*z*+1.

### Hydrogen-bond geometry (Å, °)

**Table:**

*D*—H···*A*	*D*—H	H···*A*	*D*···*A*	*D*—H···*A*
O1W—H1WB···O1^ii^	0.85	1.85	2.6891 (19)	172
O1W—H1WA···O2^iii^	0.85	1.80	2.6365 (17)	169

Symmetry codes: (ii) *x*+1, −*y*+3/2, *z*+1/2; (iii) −*x*+1, *y*−1/2, −*z*+1/2.
